# Automatic classification of diseases from free-text death certificates for real-time surveillance

**DOI:** 10.1186/s12911-015-0174-2

**Published:** 2015-07-15

**Authors:** Bevan Koopman, Sarvnaz Karimi, Anthony Nguyen, Rhydwyn McGuire, David Muscatello, Madonna Kemp, Donna Truran, Ming Zhang, Sarah Thackway

**Affiliations:** Australian e-Health Research Centre, CSIRO, Royal Brisbane and Women’s Hospital, Brisbane, Australia; NSW Ministry of Health, North Sydney, Sydney, Australia

**Keywords:** Syndromic surveillance, Machine learning, Death certificates

## Abstract

**Background:**

Death certificates provide an invaluable source for mortality statistics which can be used for surveillance and early warnings of increases in disease activity and to support the development and monitoring of prevention or response strategies. However, their value can be realised only if accurate, quantitative data can be extracted from death certificates, an aim hampered by both the volume and variable nature of certificates written in natural language. This study aims to develop a set of machine learning and rule-based methods to automatically classify death certificates according to four high impact diseases of interest: diabetes, influenza, pneumonia and HIV.

**Methods:**

Two classification methods are presented: i) a machine learning approach, where detailed features (terms, term n-grams and SNOMED CT concepts) are extracted from death certificates and used to train a set of supervised machine learning models (Support Vector Machines); and ii) a set of keyword-matching rules. These methods were used to identify the presence of diabetes, influenza, pneumonia and HIV in a death certificate. An empirical evaluation was conducted using 340,142 death certificates, divided between training and test sets, covering deaths from 2000–2007 in New South Wales, Australia. Precision and recall (positive predictive value and sensitivity) were used as evaluation measures, with F-measure providing a single, overall measure of effectiveness. A detailed error analysis was performed on classification errors.

**Results:**

Classification of diabetes, influenza, pneumonia and HIV was highly accurate (F-measure 0.96). More fine-grained ICD-10 classification effectiveness was more variable but still high (F-measure 0.80). The error analysis revealed that word variations as well as certain word combinations adversely affected classification. In addition, anomalies in the ground truth likely led to an underestimation of the effectiveness.

**Conclusions:**

The high accuracy and low cost of the classification methods allow for an effective means for automatic and real-time surveillance of diabetes, influenza, pneumonia and HIV deaths. In addition, the methods are generally applicable to other diseases of interest and to other sources of medical free-text besides death certificates.

## Background

Public health surveillance is “ongoing systematic collection, analysis and interpretation of health-related data with the a priori purpose of preventing or controlling disease or injury and identifying unusual events of public health importance, followed by the dissemination and use of such information for public health action” [1]. Death is the most severe outcome of disease or injury and is thus of fundamental significance to health surveillance. In Australia, registration of the fact and cause of death is legislated. The cause of death is recorded by a medical practitioner on the “Medical Certificate - Cause of death” [2]. The information contributes to vital statistics reporting nationally and internationally [3].

The “Medical Certificate - Cause of Death” is a medical and legal document listing, for a single person, the underlying and contributing causes leading to death. In New South Wales (NSW) cancer [4] and HIV [5] deaths are subject to regular formal reporting, for example NSW Health maintains a website which monitors the health of people in NSW [6]. However, to realise their surveillance and statistical value, cause of death information on death certificates needs to be classified and categorised. This task is typically routinely performed using computer-aided classification by expert clinical coders employed by national statistical agencies. Coronal inquiries for unnatural deaths can result in a cause of death being determined at a later date than the registration of the fact of death. These factors lead to delays of several years in the release of cause of death statistics. The coding process is hampered by the fact that the cause of death information is written in natural language and may be inconsistently structured and ambiguous. The International Classification of Diseases - Revision 10 (ICD-10) is currently used for classifying the underlying and contributing causes of death. The coding process is laborious and costly. From a surveillance perspective, government health administrations require timely information on causes of death to provide rapid assessment of disease prevention and health protection priorities. For example, organisations such as the Centre for Disease Control [7] and NSW Health [8], use cause of death data to assess the severity of pandemic and seasonal influenza in populations requires the timely reporting of deaths from pneumonia and influenza.

More timely reporting of more diverse causes of death would facilitate important feedback to health jurisdictions on the success of their disease and injury prevention programs.

In this paper, we describe a system for the automatic classification of free-text death certificates that could allow for real-time surveillance of death certificates. Two alternative approaches were developed: 1) a machine learning approach, where detailed features were extracted from the death certificate and were used to train a set of supervised classifiers; and 2) a set of keyword-spotting rules. For the machine learning approach, classification was done at two levels: disease name (‘nominal classification’) and more fine-grained ICD-10 code (e.g., E10 vs E11: insulin vs. non-insulin-dependent diabetes). (The rule-based approach could only be developed for the nominal approach). Both approaches were trialled to identify if death certificates contained any cause-of-death (not necessarily being the underlying cause-of-death) related to on the following diseases: pneumonia, influenza, diabetes and HIV.

A detailed empirical evaluation against seven years of manually coded death certificates showed that the proposed system was highly accurate at disease-of-interest classification (F-measure 0.96). Fine-grained ICD-10 classification was more challenging for an automated system but was still effective (F-measure 0.80) — less accurate results were often characterised by those ICD-10 diagnoses with little or no training data available. Furthermore, a detailed, manual analysis of the errors was conducted to gain a greater understanding of the data, classification task and areas for future work. This analysis revealed a number of areas of improvement, including handling word variants (e.g., “pneumonitis” or “pneumonic” as variants for “pneumonia”) in the death certificates, word-phrase combinations and class confusions (e.g., E10 vs. E11). In addition, this analysis highlighted anomalies in the ground truth (for example, coroner’s cases where all cause-of-death information was not recorded on the death certificate) that would lead to an underestimation of the effectiveness of the methods.

The methods proposed in the study provide an efficient and effective real-time surveillance method for a set of key diseases of interest. These methods are generally applicable to the surveillance of other diseases and may also be applicable to other data sources besides death certificates.

### Related work

Syndromic surveillance involving rapid capture, analysis and reporting of administrative data for the purpose of public health surveillance is an important part of an effective health system. Its prominence has increased in recent years with the increase in the volume and accessibility of electronic data and automated surveillance methods are increasingly researched. This has led to the development of open source systems specifically designed for outbreak and disease surveillance [9]. Naturally, much of this previous research has focused on surveillance of hospital emergency department data [10] or telephone triage data [11] as these settings are ideal for capturing data at the early stages of acute illness and may permit a rapid public health response to outbreaks. Influenza, a fast moving epidemic disease that annually causes high morbidity and mortality in populations and which has pandemic potential, is a frequent focus of syndromic surveillance [12, 13].

The previous work on disease surveillance on hospital data often made use of coded data (e.g., ICD-10 codes) [9, 14, 15] to identify particular diseases, thus the focus was not on extracting the diseases but instead on monitoring and identifying outbreaks. In contrast, the focus of this study is how to identify the disease from non-coded, free-text data — a necessary step prior to the monitoring stage. Some previous research has specifically dealt with surveillance from free-text data, both from a machine learning [10] and rule-based approach [8].

While death is the ultimate outcome of disease and its occurrence lowers urgency of the case to be investigated, it is nevertheless still a high priority for syndromic surveillance. Fatal diseases focus prevention efforts and public attention far more than milder illness. Death certificates provide an ideal administrative data source for syndromic surveillance. They have a specific format and language that differs from, for example, emergency department notes and therefore may require a different set of methods for automatic classification. Some initial work has been done on automatic classification of death certificates, from both a rule-based approach for pneumonia and influenza [8, 16] and a machine learning approach for cancer [17]. However, these studies have been small in scale and focus on one main disease and one main method (either rule-based or machine learning). The contribution of this paper is

i) methods for automatic classification of a number of different diseases (diabetes, influenza, pneumonia and HIV); ii) comparison of both rule-based and machine learning methods; iii) a large-scale empirical evaluation of the proposed methods. In addition, classification methods are developed and evaluated for both course-grained disease of interest and fine-grained ICD-10 level.

## Methods

Two alternative classification methods were investigated: a supervised machine learning approach and a rule-based approach; each are described and evaluated independently.

### Machine learning methods

We adopted a supervised machine learning method. First, detailed features were extracted from the death certificates, then ground truth labels (computer-aided human ICD-10 coding from official statistics) were assigned to each certificate and finally the labelled features were used to train a predictive model. The model was then used to classify uncoded death certificates, based on their extracted features.

#### Feature extraction methods

First, we applied a natural language processing pipeline that extracted, from a death certificate, an array of different features that could be used to train a classification model. A number of different feature types were used; these fell into two different categories: i) basic *term-based* features taken directly from the text of the death certificate; and ii) *concept-based* features, derived from the original terms, where concepts belong to standard medical terminologies (e.g., the SNOMED CT ontology). The process of extracting concepts from free-text was performed by Medtex, a clinical natural language processing system [18, 19]. Table 1 describes the different types of features extracted, belonging to these two categories. For each feature type, a description and an example of the features that were consequently derived given the text fragment is provided. The feature types listed here were chosen because they were shown to be successful in a previous study on classification of cancers from death certificates [17].

**Table 1:** Types of features — both term and concept-based — extracted from death certificates

	Feature type	Description	Example certificate extract	Resulting feature values
Term	TokenStem	A token stem, i.e., the stemmedversion of a word.	Acute chronic renal failure	Acut, chronic, renal, failur.
	TokenStem *n*-gram	The *n*-gram formed by *n* adjacenttoken stems.	chronic renal failure	Chronic renal, renal failur.
Concept	SCTConceptId	SNOMED CT concept identifier (as extracted by the Medtex system)	chronic renal failure	90688005.

(Stemming is a process of removing and replacing word suffixes to arrive at a common root form of the word.)

Once all features were extracted, death certificates were transformed from original terms to vectors of features (one vector per certificate); for example, each word (TokenStem) or SNOMED CT concept represented a single feature dimension in the vector, with features grouped into high level feature types (TokenStem or SCTConceptId). The vector comprised binary values indicating if that feature was present in the particular death certificate. Once each death certificate was represented as a feature vector, this feature vector was used as the input to the machine learning classifier.

#### Classifier model training & testing

Using the feature vectors described in the previous section, a single classifier model was trained for each of the four diseases of interest (pneumonia, influenza, diabetes and HIV) and a single classifier model for each of ICD-10 codes representing these diseases (e.g., E10, E11, E13 and E14 represented diabetes). Each model performed a binary classification on their respective class; for example, a “is diabetes” for the diabetes classifier and a “is E10” for the E10 classifier. The models were trained by dividing the death certificates according to a stratified 80/20 training/testing split (more details on experimental setup in Section ‘Data and experimental setup’). In total, 14 separate models were trained.

For the implementation of the classifiers we use Support Vector Machines (SVMs).^1^ SVMs were chosen as they were the best performing classification model in a previous death certificate classification task [17]. The Weka toolkit [20] was used for the SVM implementation. The parameters for all classifiers were set to the defaults described in Witten et al. [20].

### Rule-based methods

The rule-based approach involved the development of a set of keywords, provided in consultation with domain experts, for each disease of interest that characterise that disease.^2^ The presence of these keywords for each disease in the death certificate text would indicate whether that certificate is a positive or negative match for that particular disease.

We adapted the rule-based method suggested by Muscatello et al. [8]. in which keywords were defined for three generic categories of influenza, pneumonia, and other. In our study, we further expanded the list of diseases used in [8] to include diabetes and HIV. The rule-based system was used to classify a set of training certificates and errors from this evaluation were used to further refine the keyword list. (Keywords were considered case insensitive). In addition, a set of excluded keywords was also added. The final list of included and excluded keywords in the rule-based system is shown in Table 2.

**Table 2:** List of keywords used to identify cause of death as diabetes and HIV

Disease	Included keywords	Excluded keywords
Pneumonia	Pneumonia, Pnuemonia, Pnemonia, Pneomonia, Pneamonia, Penumonia, Pheumonia	Aapiration, Aspirare, Aspiranion
Influenza	Influenza, Influenza, H1N1, Swine Flu, Swineflu, Swine Influ, SwineInflu	Haemophilus Influenzae, Haemophilus
Diabetes	Diabetes, NIDDM, IDDM, Diabetes type 1, Diabetes Type I, Diabetes Type 2, Diabetes Type II, Type I diabetes, Type II diabetes, Type 1 diabetes, Type 2 diabetes, Type 2 diabetic mellitus, Type II diabetic mellitus, Diabetes mellitus Type 2, Diabetes mellitus Type II, Diabetic	
HIV	HIV, AIDS, human immunodeficiency	

We note that these rules are currently manually constructed and therefore they have two main restrictions: 1) they are limited to the expert’s knowledge of the terminology used to describe a particular disease, and 2) they are not automatically updated and no weighting is assigned to the keywords.

### Data and experimental setup

In this section, we detail the data used in our empirical evaluation, including development of the ground truth and the method for compiling separate training and test sets.

#### Death certificate collection and ground truth

The data consisted of de-identified death certificates covering the period 2000–2007 (340,142 certificates in total). Each certificate came with the following information:

- Free-text cause of death description, both immediate and conditions leading to death, was used as the input to our machine learning and rule-based classification methods.
- A set of ICD-10 codes representing the cause of death as determined by the Australian Bureau of Statistics. These codes represent ground truth against which our methods were evaluated. All ICD-10 codes were truncated at the three character level; for example, the code E11.1 (*Non-insulin-dependent diabetes mellitus: With ketoacidosis*) was converted to simply E11 (*Non-insulin-dependent diabetes mellitus*). Multiple ICD-10 codes could be assigned to a single certificate, however, a single code represents the *underlying* cause of death (all other codes refer to *alternative* causes of death). The four diseases of interest — pneumonia, diabetes, influenza and HIV — were identified based on a set of ICD-10 codes covering that disease. Details of this and the number of certificates associated with each ICD-10 codes is shown in Table 3.

**Table 3:** Breakdown of the dataset according to disease of interest and ICD-10 code and based on underlying and alternative cause of death numbers

Disease/ICD-10	#Underlying COD	#Alternative COD	#Total
Diabetes	7144	22647	29791
E10	830	1933	2763
E11	2449	10307	12756
E13	2	19	21
E14	3862	10387	14249
O24 ×	1	1	2
Influenza	148	44	192
J09 ×	0	0	0
J10 ×	10	3	13
J11	138	41	179
Pneumonia	7259	36688	43947
J12	33	38	71
J13	59	39	98
J14 ×	5	11	16
J15	241	405	646
J16 ×	3	6	9
J17 ×	0	0	0
J18	6918	36189	43107
HIV	371	406	777
B20	139	17	156
B21	59	6	65
B22	80	9	89
B23	54	21	75
B24	39	398	437

The diseases of interest are comprised of the sum of the individual ICD-10 codes they represent. Individual classifiers were not built for ICD-10 classes marked with a ‘ ×’ due to insufficient number of cases for these classes
- A flag representing whether the death involved a coronial inquiry. Coroner’s cases are handled differently by the clinical coders, who often have access to additional information about the death that is not stated in the death certificate. As such, we wished to identify these cases to understand the impact on system performance. The dataset contained 40,512 coroner cases (12 % of the total collection).

Pneumonia, diabetes, influenza and HIV were chosen because they are of importance to health agencies and because they cover both high prevalence diseases (Diabetes and Pneumonia) and very low prevalence diseases (Influenza and HIV). This was done to ensure that the proposed methods were evaluated on both high and low prevalence diseases.

#### Training & test split and evaluation measures

The dataset was divided into two parts: a training and test set, covering 80 % (270,742 certificates) and 20 % (68,470 certificates) of data respectively. This was done according to a random sampling stratified by underlying cause of death code. The training set was used to both train the machine learning classifiers and to develop the keywords for the rule-based approach. The test set was kept as unseen data purely for evaluation purposes. Some ICD-10 codes did not contain sufficient cases to form a 80/20 split and, therefore, classifiers were not built for these certificates; these are marked with a ‘ ×’ in Table 3.

Two evaluation measures are considered: precision and recall. Precision (also called positive predictive value) is the fraction of positively classified certificates that belong to the correct class^3^, while recall (also called sensitivity) is the fraction of actual certificates of that class that are positively classified.^4^ For disease surveillance, both precision and recall are important: a high precision indicates that the system assigns the right disease to a certificate, while a high recall indicates the system does not miss certificates that contain that disease (particularly important for rarer diseases). To provide a single, overall evaluation measure, precision and recall are combined into a third evaluation measure, F-measure.^5^

Data did not contain variables with identifying information such as names, dates of birth or addresses and ethical approval was not required.

## Results and discussion

**Fig. 1:**
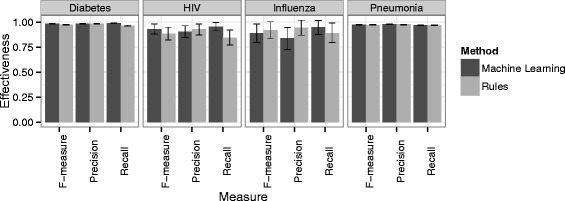
Classification performance results for diseases of interest: Influenza, Diabetes, Pneumonia and HIV. Error bars show 0.95 confidence intervals

Table 4 presents the detailed classification results for diseases of interest, with rule-based results shown in (a) and machine learning results shown in (b). In addition, a confusion matrix, which provides a breakdown of true positives, false positives, true negatives and false negatives, is shown for each disease. A graphical summary of the results is shown in the plot of Fig. 1.

**Table 4:** Classification performance results for diseases of interest: Influenza, Diabetes, Pneumonia and HIV

			(a) Rule-based				
Disease	Precision	Recall	F-measure	Confusion matrix			
				Classifier		Ground truth	
				-	+		
Influenza	0.94	0.89	0.92	68430	2	-	
				4	34	+	Influenza
Pneumonia	0.98	0.97	0.97	59351	215	-	
				274	8630	+	Pneumonia
Diabetes	0.98	0.96	0.97	62,519	100	-	
				212	5639	+	Diabetes
HIV	0.93	0.85	0.89	68,373	6	-	
				14	77	+	HIV
Macro-average^*a*^	0.94	0.96	0.95				
Micro-average^*b*^	0.98	0.98	0.98				
			(b) Machine learning				
Disease	Precision	Recall	F-measure	Confusion matrix			
				Classifier		Ground truth	
				-	+		
Influenza	0.84	0.95	0.89	68425	7	-	
				2	36	+	Influenza
Pneumonia	0.98	0.97	0.97	59364	202	-	
				279	8625	+	Pneumonia
Diabetes	0.98	0.99*	0.99*	62522	97	-	
				72	5779	+	Diabetes
HIV	0.91	0.96	0.93	68370	9	-	
				4	87	+	HIV
Macro-average	0.93	0.97	0.94				
Micro-average	0.98	0.98	0.98				

^*a*^Macro-average is the mean of the precision, recall, and f-measure values from the four classes above

^*b*^Micro-average aggregates the values from the confusion matrix for all the classes and calculates the measures over all the data

Statistically significant differences between rules and machine learning as measured with a two-tailed z-test are marked with *, representing *p*<0.05

Both methods demonstrated good performance across the different diseases. The two methods had comparable performance, with the only statistically significant difference between the two being on diabetes, where the machine learning method was superior. For influenza, the rule-based approach was more effective, likely because it is the smallest class in terms of the amount of training and testing data which adversely influences the effectiveness of machine learning methods. For HIV, the machine learning method is more effective, likely because the machine learning method effectively accounted for the many different variants of describing HIV (AIDS, HIV, Human Immunodeficiency Virus). (However, a larger sample size would be required to determine statistical significance for influenza and HIV). Overall, both methods have higher recall than precision, showing that false positive errors are more common than false negative errors. The fact that recall is higher than precision may be more reflective of disease prevalence. The prevalence of diabetes and pneumonia are high but the prevalence of HIV and influenza are very low; this accentuates the effect of false positives on recall. It is important to note that the methods proposed here are effectives across a range of both high and low prevalence diseases. 

Table 5 presents the results for ICD-10 classification (machine learning only). Compared with the disease of interest results, the ICD-10 classification demonstrated more variable results: most models were highly effective (E10, E11, E14, J11, J12 and J18), while some others were less effective (E13, J13 and J15). Poor performance was generally characterised by rarer ICD-10 codes: those with only a small number of cases (shown as the sum of the bottom row of the confusion matrix for each ICD-10 code). This is also demonstrated by the difference between the macro-average (the mean of the individual performances for each classifier) and the micro-average (the sum of the individual true positives, false positives, and false negatives, divided by the total number of cases); the lower performance of rarer ICD-10 classes reduces the macro-average but only contributes a small number of errors toward the overall micro-average.

**Table 5:** Classification performance results for individual ICD10 classes

	Disease	Precision	Recall	F-measure	Confusion matrix			
					Classifier		Ground truth	
					-	+		
Diabetes	E10	0.76	0.97	0.86	67774	162	-	
					14	520	+	E10
	E11	0.97	0.97	0.97	65852	89	-	
					78	2451	+	E11
	E13	0.40	0.50	0.44	68463	3	-	
					2	2	+	E13
	E14	0.96	0.97	0.96	65521	116	-	
					97	2736	+	E14
Flu	J11	0.88	0.86	0.87	68431	4	-	
					5	30	+	J11
Pnuemonia	J12	1.00	0.93	0.97	68455	0	-	
					1	14	+	J12
	J13	0.79	0.55	0.65	68447	3	-	
					9	11	+	J13
	J15	0.92	0.35	0.51	68331	4	-	
					88	47	+	J15
	J18	0.97	0.97	0.97	59480	244	-	
					286	8460	+	J18
Macro-average		0.85	0.78	0.80				
Micro-average		0.96	0.96	0.96				

While both methods — rule and machine learning — had comparable overall effectiveness, they have different advantages and disadvantages. The rules have to be manually defined so adding additional diseases requires manual intervention. The machine learning classifiers do not require manual intervention; however, they do require that suitable labelled training data is available (which may require machine intervention). The rules are computationally very simple: deployed easily and useing very little computational resources. The machine learning methods require a suitable pipeline to extract features from the death certificates and then train an appropriate support vector machine, some of which can be computationally expensive for large collections. Thus the two methods could be seen as complimentary given the individual application to which they may be applied.

Comparing the results here with those of previous studies outline in the Related Work, the rule-based results for Influenza and Pneumonia were inline with that of Muscatello et al. [8]. (HIV and Diabetes were not considered in that previous study). For the machine learning results, comparison is made against the classification methods of Butt et al. [17], who applied similar techniques to identifying the presence of cancer from death certificates. F-measure results were 0.98 — the same as the machine learning methods reported in this study. It is worth noting though that the tasks differ somewhat between the two studies: i) cancer is a broad range of different conditions, whereas influenza, diabetes, pneumonia and HIV are more specific ii) the task was predict cancer as the *underlying* cause-of-death, whereas as the task here was to predict influenza, diabetes, pneumonia and HIV as any contributing cause-of-death.

### Error analysis

While the disease of interest classification was highly effective, the ICD-10 classification demonstrated some variable results. To further understand the issues and factors influencing ICD-10 classification a detailed manual review of classification errors was undertaken. A subset of 495 incorrectly classified death certificates were reviewed by two authors with clinical coding experience (DT & MK). The reasons underlying the errors were identified and errors were assigned to one or more categories (the breakdown of which is shown in Table 6):Word variations: Lexical variants of the same disease. For example, “pneumonitis” or “pneumonic” as variants for “pneumonia”; or “type II” or “type two” to express type 2 diabetes. Also, included are some misspellings, e.g., “aspiranion” and “phenomia”. Word combinations: Words in combination create a phrase with an alternative meaning than the individual words in isolation and, therefore, an alternative cause-of-death. For example, the word combination “diabetes insipidis”, which, although containing the word “diabetes” is, in fact, a separate condition and should be assigned E23 (*Hypofunction and other disorders of pituitary gland*) not E14 (*Diabetes mellitus*). Word combinations were a feature extracted from the death certificate (see the TokenStem n-gram feature from Table 1); however, there was likely insufficient samples of these cases for training a model that was sensitive to such cases. Secondary causes: A number of false negatives were observed where the disease of interest was found in Section II of the death certificate. Section II is defined as “Other significant conditions contributing to the death, but not related to the disease condition causing it”, while Section I is “Disease or condition directly leading to death”. For these cases, the presence of the entries in Section I would have likely led the classifier to assign a negative label to the certificate. Class confusion: A number of errors resulted from confusion between the specific codes for a particular disease; for example, confusion between *Insulin-dependent diabetes mellitus* (E10), *Non-insulin-dependent diabetes mellitus* (E11) and *Unspecified diabetes mellitus* (E14); similarly, between *Bacterial pneumonia* (J15) and *Pneumonia, organism unspecified* (J18). The source of class confusion is primarily in that the two codes (diseases) are difficult to distinguish from each other (for an automated classifier). For example, the feature vectors for three death certificates containing “Type I diabetes” (E10), “Type II diabetes” (E11) and “diabetes” (E14) are very similar to each other. Thus, it is difficult for the machine learning method to differentiate between them. In addition, there are multiple ways to express the same disease; e.g., for Diabetes, “insulin-dependent” or “Type I” (E10) and for Pneumonia, “bacterial” or “streptococcal” (J15). This means that the feature vectors belonging to a single class (e.g., all the J15s) are quite different from each other; again making it different for the machine learning method to accurately differentiate from other classes. Ground truth class confusion: There were instances where the ground truth label did not appear accurate (according to the ICD-10 cause of death coding guidelines [21]). For example, a death certificate containing “pneumonia, aspiration” coded with J18 rather than J69 (*Pneumonitis due to solids and liquids*); or “diabetes (type II)” coded with E14 (unspecified) rather than E11 (non-insulin-dependent). Ground truth error: Prediction appears correct based on the available text, however the ground truth contained a number of additional codes, leading to the assumption that further information was available to the clinical coders which was not evident in the text. This is often the case for coronial inquiries, where the clinical coder has access to additional cause of death information from the coronial information system. For example, a death certificate containing only the text *Multiple Injuries* but coded with J18 (*Pneumonia, organism unspecified*). Ground truth empty: For a small number of death certificates, no ground truth codes were available.

**Table 6:** Breakdown of classification errors according to different error categories

Category	Total #errors	% of total	#coroner
		records	cases
Classification errors:	405	58.0	76
Word variations	75	11.5	12
Word combinations	98	13.0	15
Secondary causes	150	22.4	43
Class confusion	82	11.1	6
Ground truth issues:	290	42.0	130
Ground truth class confusion	98	12.7	10
Ground truth error	167	25.5	113
Ground truth empty	25	3.8	7

Categories are divided into actual classification errors and other issues related to the use of ICD-10 codes as the ground truth label

Table 6 shows that 42 % of errors were from categories that were related to ground truth issues; the most significant being the Ground truth error category predominantly the result of death certificates involving coronial inquiries where additional information was available to the clinical coder. These certificates could be excluded — either not used in training the model or excluded from the empirical evaluation — however, it is valuable to understand the effect that such certificates had on classification effectiveness. Overall, the issues related to ground truth would suggest that the effectiveness of the classification methods was underestimated and that the actual classification effectiveness for ICD-10 would be higher.

### Limitations and future work

A limitation of the proposed methods is that adding new diseases requires the development of new models and rules. For the machine learning methods, this is done by simply training a new model, provided the labelled ground truth data is available. Developing new rules is more laborious as it requires manually analysing certificates to identifying relevant keywords for the new rules.

Changes in different diseases may also affect the performance of the classifiers over time. For example, in this study, death certificates were within the 2000–2007 timeframe and, therefore, did not contain deaths from the H1N1 pandemic of 2009. Thus, the methods would likely not identify a H1N1 death as being a Influenza related. Retraining or incrementally updating the classifiers would be required to keep them up-to-date with changes in diseases.

In this study, only the textual cause-of-death entry was available to us and used to classify death certificates. However, a death certificate does contain addition information, including place of death (home, hospital, etc.), age, gender and whether the cause was a coronial case. This information may be valuable to include as additional features for the classification methods. Certainly, the previous section highlighted issues related to coronial cases that could likely be alleviated if this information was included as additional features.

A number of areas of future work arise from the error analysis. Given that the vocabulary of death certificates is somewhat constrained, an effective term normalisation (e.g., folding synonyms to a single root term) method could be developed to deal with issues around word variations. This method may also help to alleviate some of the issues around word combinations: normalising terms would also result in normalisations of phrases, thus providing more training samples for such phrases. More errors were observed when diseases of interest were mentioned in Section II of the death certificate^6^; thus, incorporating section information into the classification system may help to alleviate such errors. Some of the issues around class confusion may be addressed by incorporating additional higher-level features (e.g., virus or bacteria type features when encountering pneumonia) with each disease. Finally, misclassification of coroner’s cases highlights a need for such cases to be handled separately by the classification system.

## Conclusions

Our study proposed and evaluated a means to automatically identify and characterise pneumonia, diabetes, influenza and HIV from large collections of free-text death certificates. This could be be implemented in the context of real-time monitoring and surveillance of mortality due to these diseases of interest. Two alternative approaches were developed: 1) a machine learning approach, where discriminating features (both term and concept-based) were extracted from the death certificate and were used to train a set of supervised classifiers, both for course-grained disease of interest and fine-grained ICD-10 causes of death; and 2) a set of keyword-matching rules at disease of interestlevel.

Empirically, disease of interest classification was highly accurate with 0.96 F-measure, while ICD-10 classification was variable but still effective with 0.80 macro-average F-measure. A detailed error analysis revealed a number of issues related to incorrect or differing ground truth — the results being that the actual effectiveness of the ICD-10 classification methods was higher than estimated. In addition, the error analysis revealed a number of areas of future work in terms of normalisation, section handling and additional higher-level features (e.g., virus vs. bacteria).

The methods and findings of this study are generally applicable to other diseases besides pneumonia, diabetes, influenza and HIV investigated here. In addition, the methods and findings are also applicable to other sources of medical free-text besides death certificates.

## Endnotes

^1^ A Support Vector Machine is a discriminative classifier formally defined by a separating hyperplane. In other words, given labeled training data (supervised learning), the algorithm outputs an optimal hyperplane which categorizes new examples.

^2^ The rule-based approach was applied to only the disease of interest and not for individual ICD-10 codes.

^3^ Precision = True Positives/(True Positives + False Positives).

^4^ Recall = True Positives/(True Positives + False Negatives).

^5^ F-measure = 2 *(Precision * Recall)/(Precision + Recall).

^6^ Section II is defined as “Other significant conditions contributing to the death, but not related to the disease condition causing it”.

## Appendix: Word variants

Table 7 provides a list of common word variants identified during the manual analysis of classification errors.

**Table 7:** Common word variants identified during the manual analysis of classification errors

Pneumonia	Influenza	Diabetes	HIV
Bronchopneumonia	influenzal	Non insulin	Acquired
			immunodeficiency
			syndrome
Pneumonitis	Type A	Non-insulin	Immune
			deficiency
			syndrome
Pneumonic	Type B	Diabetic	Human
			immunosuppressive
			virus
Broncho-pneumonia	Parainfluenza	DM	Human immuno
			deficiency virus
Bronchopneumonitis	Haemophilus	IDD	
Pneumocystis	Haemophyllis	IDDI	
	influenzae		
	Influenza A		
	Influenza B		
	Parainfluenza III		
	High influenza		
	Influenza-like		
